# Improving the accuracy of Aboriginal and non-Aboriginal disease notification rates using data linkage

**DOI:** 10.1186/1472-6963-8-118

**Published:** 2008-05-30

**Authors:** Donna B Mak, Rochelle E Watkins

**Affiliations:** 1 Communicable Diseases Control Directorate, Western Australian Department of Health, Perth, Australia; 2 Australian Biosecurity CRC, Faculty of Health Sciences, Curtin University of Technology, Perth, Western Australia, Australia

## Abstract

**Background:**

Routinely collected infectious disease surveillance data provide a valuable means to monitor the health of populations. Notifiable disease surveillance systems in Australia have consistently reported high levels of completeness for the demographic data fields of age and sex, but low levels of completeness for Aboriginality data. Significant amounts of missing data associated with case notifications can introduce bias in the estimation of disease rates by population subgroups. The aim of this analysis was to evaluate the use of data linkage to improve the accuracy of estimated notification rates for sexually transmitted infections (STIs) and blood borne viruses (BBVs) in Aboriginal and non-Aboriginal groups in Western Australia.

**Methods:**

Probabilistic methods were used to link disease notification data received in Western Australia in 2004 with core population health datasets from the established Western Australian Data Linkage System. A comparative descriptive analysis of STI and BBV notification rates according to Aboriginality was conducted based on the original and supplemented notification datasets.

**Results:**

Using data linkage, the proportion of STI and BBV notifications with missing Aboriginality data was reduced by 74 per cent. Compared with excluding notifications with unknown Aboriginality data from the analysis, or apportioning notifications with unknown Aboriginality based on the proportion of cases with known Aboriginality, the rate ratios of chlamydia, syphilis and hepatitis C among Aboriginal relative to non-Aboriginal people decreased when Aboriginality data from data linkage was included.

**Conclusion:**

Although there is still a high incidence of STIs and BBVs in Aboriginal people, incompleteness of Aboriginality data contributes to overestimation of the risk associated with Aboriginality for these diseases. Data linkage can be effectively used to improve the accuracy of estimated disease notification rates.

## Background

Infectious disease notification data provide a valuable means to monitor the health of populations and indicate priorities for health policy, resource allocation and disease control. High levels of reporting completeness for diagnosed cases recorded in notifiable diseases surveillance systems is essential for these systems to adequately inform disease prevention and control activities [1]. The item-level completeness of case notifications also has the potential to impact on the usefulness of routinely collected notifiable disease data for population health. Missing socio-demographic identifiers for notified cases can compromise the validity of inferences based on the data by introducing bias in estimated disease notification rates by population subgroups.

Despite medical practitioners in Western Australia having a statutory obligation to provide data on a patient's Aboriginality to the Department of Health when a patient is diagnosed with a notifiable infectious disease, notifiable disease data for Western Australia have a limited capacity to identify Aboriginality. Historically, annual reports of Australia's notifiable disease surveillance system have not included Aboriginality in the analyses due to incomplete reporting of this information [2]. The most recent national-level report describing Australian notifiable disease data found that Aboriginality was complete in only 50 per cent of notifications in 2005 [3]. The level of completeness of Aboriginality in the 2005 notification dataset reflects a continuing trend of improving completeness, with only 46 per cent of national notifications in 2004 [4] and 43 per cent of national notifications in 2003 [5] being complete for Aboriginality. In comparison, 99.9 per cent of national notifications in 2005 were complete for sex, and 99.8 per cent were complete for date of birth [3].

Based on the 2006 census, the Aboriginal and Torres Strait Islander population of Australia is estimated to exceed half a million people [6]. Although the Aboriginal and Torres Strait Islander population only represents 2.5 per cent of the Australian population, the excess rates of morbidity and mortality among Aboriginal people in Australia are a significant population health issue [7,8]. The Aboriginal population in Australia are more disadvantaged in term of excess mortality than the indigenous populations of Canada, New Zealand or the United States [7], and these deaths are principally from preventable causes including heart disease, smoking-related diseases, injury and specific types of cancer [9]. Following adjustment for age, Aboriginal people in Australia have a higher prevalence of most types of health conditions, a higher prevalence of many notifiable communicable diseases, and twice the hospitalisation rate when compared with non-Aboriginal people [10].

Due to the high proportion of missing data on Aboriginality, it has been argued that the disease notification rates calculated from the national notifiable disease data underestimate the true rates of disease among Aboriginal people, independent of other biases that influence the likelihood of disease being identified and diagnosed [11]. An understanding of the association between item non-response and Aboriginality, and the extent that this differs from the association between Aboriginality and disease among the observed notification data is required to determine if non-response bias exists in the estimation of disease notification rates by Aboriginality.

In practice, cases for which there is no information on Aboriginality are often excluded from calculations of disease rates by Aboriginality. As such, the reported disease rates may be inaccurate, particularly if Aboriginality is unknown in a substantial proportion of cases. As an alternative to excluding cases with missing data in the estimation of disease notification rates, health authorities have also estimated rates by apportioning notifications with unknown Aboriginality to the Aboriginal and non-Aboriginal categories according to the proportions observed among the non-missing data. This method assumes that cases with unknown Aboriginality have a comparable distribution of Aboriginality to cases with known Aboriginality. However, there is little evidence to suggest that the available data are an unbiased indicator of disease rates by Aboriginality.

Neither of these commonly used methods is ideal for the estimation of disease rates by Aboriginality in the presence of substantial amounts of missing data, as both may lead to significantly biased estimated rates. As disease rates based on case notification data are often used as the basis for policy and funding decisions, it is important for these rates to be calculated as accurately as possible.

Although methods for the linkage of data within public health agencies to inform health policy are well established [12], data linkage is generally not routinely performed to improve the quality of infectious disease surveillance data. To date there has been no systematic exploration of the contribution of missing data on Aboriginality to inaccuracy in the estimated notification rates of sexually transmitted infections (STIs) and blood borne viruses (BBVs) by Aboriginality in Western Australia. The aim of this analysis was to evaluate the use of data linkage to improve the accuracy of estimated notification rates for STIs and BBVs in Aboriginal and non-Aboriginal groups in Western Australia.

## Methods

### Data sources and data linkage

All notified cases of STIs and BBVs (excluding HIV cases which are notified separately and for which Aboriginality data are complete) in Western Australia with a case report date between the 1^st ^January 2004 and the 31^st ^December 2004 were extracted from the Western Australian Notifiable Infectious Diseases Database for analysis. Data extracted included case notification data for chlamydia, gonorrhoea, syphilis (primary, secondary, tertiary and latent), hepatitis B and hepatitis C. We used established linked administrative health databases to obtain information on Aboriginality where this information was missing in the notifiable diseases dataset. In this paper, the term Aboriginal is used in preference to 'Aboriginal and Torres Strait Islander' in recognition that Aboriginal people are the original inhabitants of Western Australia.

As the Australian health system does not use unique identification numbers to record individual health service encounters, probabilistic methods were used to identify linkages between health records. The demographic details (surname, first name, date of birth, sex and residential address) of all 2004 notifications for STIs and BBVs that were of unknown Aboriginality were extracted and used to identify linked records in the Western Australian Data Linkage System [12]. The Western Australian Data Linkage System systematically links administrative health data in Western Australia from core administrative health databases. The record linkages are created and maintained using rigorous internationally accepted privacy-sensitive protocols, probabilistic matching based on multiple data fields (unit medical record number, surname, first name, initial, sex, date of birth and residential address) and name compression algorithms, multiple matching passes, and extensive clerical review of all potential linkages that are not identified as definite matches. The system is described in detail in Holman et al. [12].

Linkage of records from the Western Australian Notifiable Diseases Database with existing linked data within the Western Australian Data Linkage System was performed by experienced staff at the Data Linkage Unit of the Department of Health, Western Australia. All available data for the following statutory datasets that are maintained by Western Australian government Departments were searched within the Western Australian Data Linkage System to determine the cases' Aboriginality: Mortality Records, Hospital Morbidity Data System, Western Australian Midwives' Notification System, and the Mental Health Information System. The Mortality Records contain a record of all deaths registered in the state of Western Australia. The Hospital Morbidity Data System contains in-patient discharge summary data from all public and private hospitals in Western Australia dating back to 1970. The Western Australian Midwives' Notification System contains midwives notifications of births occurring in Western Australia since 1980. The Mental Health Information System collects information about people who utilise mental health services in Australia including both hospital inpatient data, and ambulatory data (non-inpatient data) from psychiatric clinics, community health centres, day centres, outreach programs and rehabilitation programs. The Mental Health Information System is the oldest continuous mental health information system in Australia, which has been operating since July 1966. These core databases are statutory databases, and have a high coverage of the Western Australian population.

Linkage of records from the Western Australian Notifiable Diseases Database with existing linked data within the Western Australian Data Linkage System used routine probabilistic matching methods to link demographic information from notification data (surname, first name, sex, date of birth and residential address) using multiple passes based on different subsets of demographic indicators. For each matching variable, weights are assigned to the record pairs to indicate the likelihood of a linkage, and the weights allocated in each pass are analysed to divide record pairs into 'definite links', 'definite non-links' and 'possible links'. Definite links are accepted and loaded, and definite non-links are discarded. All possible links were subject to clerical review by highly specialised Linkage Officers who have access to the historical archive of previously linked records and determine whether the available evidence supports the creation of a link or not.

### Assignment of Aboriginality

**Figure 1:**
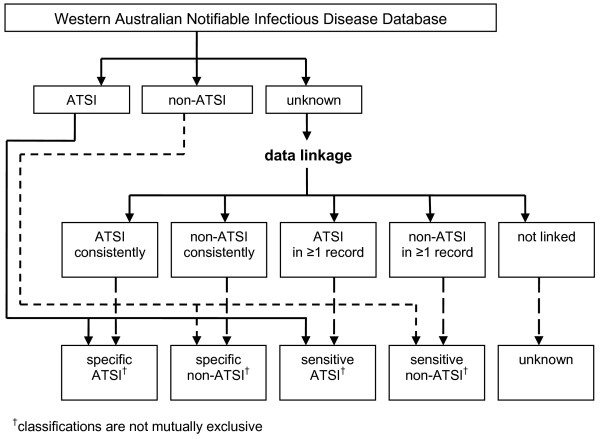
Summary of the process used to derive sensitive and specific indicators of Aboriginality using data linkage.

Data on Aboriginality obtained via data linkage were combined with data on Aboriginality from the Western Australian Notifiable Infectious Disease Database to derive both 'sensitive' and 'specific' indicators for Aboriginal and non-Aboriginal status respectively, as summarised in Figure 1.

The sensitive definition of Aboriginal included a person who was identified as Aboriginal or Torres Strait Islander (ATSI) on the case notification form, or if Aboriginality data were missing from the notification, a person who was identified as ATSI at least once via the data linkage process. The specific definition of Aboriginal includes a person who was identified as ATSI on the case notification form, or if Aboriginality data were missing from the notification, a person who was identified consistently (one or more times) as ATSI via the data linkage process. If an individual was only identified once via the data linkage process as ATSI, this identification was considered consistent, and the individual was included in the specific definition of Aboriginal. Thus, the sensitive definitions differ from the specific definitions only in cases where more than one linked health record exists, and the information contained in these linked records is not consistent.

The sensitive definition of non-Aboriginal included a person who was identified as non-ATSI on the case notification form, or if Aboriginality data were missing from the notification, a person who was identified as non-ATSI at least once via the data linkage process. The specific definition of non-Aboriginal includes a person who was identified as non-ATSI on the case notification form, or if Aboriginality data were missing from the notification, a person who was identified consistently (one or more times) as non-ATSI via the data linkage process.

Persons who had Aboriginality data missing from the case notification, and who were not identified during the linkage process were excluded from the sensitive or specific definitions of Aboriginality. The classification rules for Aboriginality allow some overlap between the sensitive definitions of Aboriginality if an individual was identified during data linkage as both ATSI and non-ATSI in more than one linked health record. Thus, the classifications used are not mutually exclusive.

### Data analysis

Data were de-identified following data linkage and coding of Aboriginality. Age-standardised disease notification rates were calculated based on the following indicators of Aboriginality: the four sensitive and specific indicators of Aboriginality derived using data linkage; the original un-linked notifiable disease data, by excluding all cases of unknown Aboriginality; and the original un-linked notifiable disease data, by apportioning notifications with missing data using the same proportions as those observed among notifications where Aboriginality was not missing in each age stratum (proportional method). Age-standardised rates were calculated using data from the 2001 census [13] using seven 10-year age strata from 0–9 years through to 60 or more years. Both numerator and denominator data used to calculate age-adjusted rates excluded persons of unknown Aboriginality.

To quantify the differences in age-standardised notification rates by Aboriginality, Aboriginal: non-Aboriginal rate ratios and their 95 per cent confidence intervals were calculated using PROC GENMOD (SAS version 9.1). A main effects Poisson regression model of the counts of notified cases was constructed for each STI and BBV, for each of the four definitions of Aboriginality: pre-linkage, proportional assignment, sensitive and specific. The model included indicator variables for Aboriginality and age-group (0–9, 10–19, 20–29, 30–39, 40–49, 50–59, 60+), and the log of the population estimates for each age stratum by Aboriginality as an offset. The model scale parameter was estimated using the deviance method to allow for over-dispersed data [14]. Rate ratios of the notification rates among Aboriginal and non-Aboriginal persons and 95 per cent confidence intervals were calculated based on the coefficients and standard errors of the Poisson regression model.

Multiple logistic regression was also used to analyse the association between whether the cases with unknown Aboriginality could be linked via the data linkage process and the case's age, gender, disease, and the location of the reporting public health unit (metropolitan or rural). All bivariate associations between the independent and dependent variables were investigated, and the final multivariate model was derived using backward manual stepwise elimination of variables.

Data were analysed using SPSS (version 12.0, SPSS Inc.), SAS (version 9.1, SAS Institute Inc.) and Microsoft Excel. The project was granted ethics approval from the Western Australian Aboriginal Health Information and Ethics Committee and the Confidentiality of Health Information Committee.

## Results

In Western Australia a total of 7619 notifications of STIs and BBVs were received in 2004, and data on Aboriginality was missing (recorded as 'unknown') in 26% of these notifications (Table 1). Chlamydia, the most commonly notified disease, had the highest proportion of cases with missing Aboriginality data. Information on Aboriginality could be determined via data linkage for 1440 (74%) of the 1959 STI and BBV (excluding HIV) notifications received in 2004 with missing Aboriginality data (Table 1). The proportion of records with missing Aboriginality that was able to be determined using data linkage varied from 53% for hepatitis B notifications to 80% for hepatitis C notifications. After data linkage, there were negligible proportions of gonorrhoea and syphilis notifications, and less than 10% of chlamydia, hepatitis B and hepatitis C with missing Aboriginality data.

**Table 1:** Aboriginality of STI^† ^and BBV^‡ ^notifications received in 2004 prior to data linkage, and the proportion of notifications with unknown Aboriginality following data linkage

	Pre-linkage			Post-linkage
		
Disease	Aboriginal n (%)	Non-Aboriginal n (%)	Unknown n (%)	Unknown n (%)
Chlamydia, n = 4329	1078 (25)	1789 (41)	1462 (34)	402 (9.3)
Gonorrhoea, n = 1433	1085 (76)	307 (21)	41 (3)	12 (0.8)
Syphilis, n = 206	137 (66)	45 (22)	24 (12)	2 (1.0)
Donovanosis, n = 1	1 (100)	0 (0)	0 (0)	0 (0)
Hepatitis B, n = 435	61 (14)	317 (72)	57 (13)	27 (6.2)
Hepatitis C, n = 1215	134 (11)	706 (58)	375 (31)	76 (6.3)

Total, n = 7619	2496 (33)	3164 (41)	1959 (26)	519 (6.8)

^† ^Sexually Transmitted Infections (STI)

^‡ ^Blood borne virus (BBV) notifications, excluding HIV (Human Immunodeficiency virus)

The 1440 linked case notification records were most frequently linked to the hospital morbidity database (99%), followed by the mental health database (29%), midwife database (18%) and death records (0.02%). Of the 1440 linked records, 61% were only linked with one database, 30% were linked with two databases, 9% were linked with three databases, and 0.002% were linked with all four databases. The linkage of case notifications with the morbidity database was frequently based on multiple records of hospital admission. Of the 1428 linkages of case notifications with the morbidity database, 337 were linked with 1 morbidity record, 260 were linked with 2 morbidity records, 192 were linked with 3 morbidity records, 121 were linked with four morbidity records and 518 were linked with 5 or more morbidity records. In 49 cases the information on Aboriginality within the morbidity database was internally inconsistent, being equivalent to approximately 4% of notifications that were found to have more than one record in the mortality database. The difference between the number of notifications that meet the sensitive and specific definitions of Aboriginality that are summarised by disease in Table 2 represent those notifications that were found to have inconsistent records for Aboriginality following data linkage.

**Table 2:** Comparison of the Aboriginality of STI^† ^and BBV^‡ ^notifications received in 2004 prior to and following data linkage by estimation method

	Aboriginal n (%)			non-Aboriginal n (%)		
		
Disease	Pre-linkage	Sensitive	Specific	Pre-linkage	Sensitive	Specific
Chlamydia	1078 (25)	1161 (27)	1127 (26)	1789 (41)	2800 (65)	2766 (64)
Gonorrhoea	1085 (76)	1093 (76)	1086 (76)	307 (21)	335 (23)	328 (23)
Syphilis	137 (66)	138 (67)	137 (67)	45 (22)	67 (33)	66 (32)
Hepatitis B	61 (14)	64 (15)	64 (15)	317 (72)	344 (79)	344 (79)
Hepatitis C	134 (11)	153 (13)	138 (11)	706 (58)	1001 (82)	986 (81)
Total	2496 (33)	2610 (34)	2553 (34)	3164 (41)	4547 (60)	4490 (59)

^† ^Sexually Transmitted Infections (STI) excluding Donovanosis

^‡ ^Blood borne virus (BBV) notifications, excluding HIV (Human Immunodeficiency virus)

Among the 1959 case notifications that were originally of unknown Aboriginality, multivariate logistic regression analysis showed that after controlling for age, the likelihood of being able to determine Aboriginality via data linkage was significantly and independently associated with sex and disease. The Aboriginality of females with missing Aboriginality data was more likely to be able to be determined via data linkage than that of males. Similarly, the Aboriginality of cases of chlamydia, syphilis and hepatitis C with missing Aboriginality data was more likely to be able to be determined via data linkage than that of cases with hepatitis B (Table 3).

**Table 3:** Multivariate analysis of association between whether Aboriginality could be determined via data linkage and the independent variables, age, sex and disease

Independent variables	p	Odds Ratio	95% CI^†^
Age (years)	0.41	1.00	0.99–1.02
Sex (female)	<0.001	1.50	1.21–1.85
Disease (reference: Hepatitis B)	<0.001	-	-
Chlamydia	0.005	2.23	1.27–3.93
Gonorrhoea	0.07	2.27	0.95–5.43
Syphilis	0.004	9.66	2.06–45.3
Hepatitis C	<0.001	3.48	1.92–6.28

^† ^Confidence Interval (CI)

### Estimated disease notification rates

Compared with the use of notification data alone, the proportion of chlamydia and hepatitis C notifications that occurred in non-Aboriginal people increased by 59% (from 41% to 65%) and 40% (from 58% to 82%) respectively when the sensitive definition of non-Aboriginal was used (Table 2). There was little difference in the proportion of STI and BBV notifications occurring in Aboriginal and non-Aboriginal people depending on whether the sensitive or the specific definitions of Aboriginality were applied.

Notification rates calculated by excluding cases where Aboriginality data were missing from case notifications often underestimate the notification rates generated using the linked data among both Aboriginal and non-Aboriginal people (Table 4). The calculation of notification rates by apportioning notifications with unknown Aboriginality using the proportional method preserves the rate-ratios observed in the pre-linkage data, and also overestimates the notification rates generated using the linked data among Aboriginal people. The age-adjusted rates of chlamydia (2269 per 100,000 population), gonorrhoea (1227 per 100,000 population), syphilis (361 per 100,000 population), hepatitis B (131 per 100,000 population), and hepatitis C (333 per 100,000 population) for Aboriginal people based on the proportional method all exceed the estimated age-adjusted rates based on the sensitive and specific definitions of Aboriginality.

**Table 4:** Comparison of age-standardised notification rates (per 100,000 population) and rate ratios of STI^† ^and BBV^‡ ^notifications received in 2004 by Aboriginality prior to and following data linkage by estimation method

	Pre-linkage			Sensitive			Specific		
			
Disease	ATSI	non-ATSI	RR (95% CI)	ATSI	non-ATSI	RR (95% CI)	ATSI	non-ATSI	RR (95% CI)
Chlamydia	1501	105	14.4 (11.2–18.6)	1608	165	9.9 (7.6–12.9)	1561	163	9.7 (7.4–12.7)
Gonorrhoea	1524	18.0	89.4 (51.0–157)	1534	19.6	82.1 (49.7–136)	1526	19.2	83.5 (49.4–141)
Syphilis	313	2.6	105 (65.3–168)	315	3.8	70.5 (44.4–112)	313	3.8	71.2 (45.1–112)
Hepatitis B	117	18.5	5.9 (4.3–8.0)	122	20.1	5.7 (4.4–7.5)	122	20.1	5.7 (4.4–7.5)
Hepatitis C	232	41.1	6.1 (4.7–8.0)	271	58.2	5.0 (4.0–6.3)	240	57.4	4.6 (3.5–6.0)

^† ^Sexually Transmitted Infections (STI) excluding Donovanosis

^‡ ^Blood borne virus (BBV) notifications, excluding HIV (Human Immunodeficiency virus)

ATSI: Aboriginal and Torres Strait Islander

RR: rate ratio estimate based on Poisson regression coefficients

CI: confidence interval

Following data linkage, the rate ratios for chlamydia and syphilis by Aboriginality decrease by approximately 30% if Aboriginality data from data linkage are included when calculating age-standardised disease notification rates (Table 4). The rate ratios for hepatitis C and gonorrhoea by Aboriginality also showed a slight decrease following data linkage compared with pre-linkage estimates. Despite the observed decrease in disparity between the age-adjusted rates of STIs and BBVs among Aboriginal and non-Aboriginal people when the completeness of Aboriginality data are improved, the rate of STIs and BBVs for Aboriginal people remained significantly higher than rates for non-Aboriginal people for all diseases examined.

## Discussion

To our knowledge this study is the first to investigate the application of data linkage methods for the improvement of routine notifiable disease surveillance analyses in Australia. Using data linkage, the proportion of STI and BBV notifications with missing Aboriginality data was able to be substantially reduced. Following data linkage, there were negligible proportions of gonorrhoea and syphilis notifications, and less than 10 per cent of chlamydia, hepatitis B and hepatitis C notifications with missing Aboriginality data. Data linkage is a useful tool for improving the completeness of Aboriginality data, and allows the calculation of more accurate STI and BBV disease notifications rates.

Notification rates calculated by excluding cases where Aboriginality data were missing commonly underestimated disease rates in both Aboriginal and non-Aboriginal people. However, disease rates in Aboriginal people were over-estimated when notification rates were calculated by apportioning notifications with unknown Aboriginality to the Aboriginal and non-Aboriginal categories using the same proportions as those observed among notifications where Aboriginality was known. This occurs because notifications with missing Aboriginality data are more likely to be identified as non-Aboriginal via data linkage. Apportioning notifications with unknown Aboriginality to the Aboriginal and non-Aboriginal categories based on the proportions observed among notifications where Aboriginality is known will result in biased estimates of disease rates among Aboriginal people, as case notifications with unknown Aboriginality systematically differ from case notifications with known data with respect to Aboriginality.

Following data linkage, the risk of STIs and BBVs associated with Aboriginality was found to decrease. Although there is a very high incidence of STIs and BBVs in Aboriginal people, and following data linkage the significant difference between disease notification rates according to Aboriginality remained; our findings suggest that Aboriginality is most probably not as strong a risk factor for chlamydia, syphilis and hepatitis C as is suggested by the notification data when cases with unknown Aboriginality are excluded.

Our estimated age-adjusted rate ratios for chlamydia and syphilis for Aboriginal versus non-Aboriginal persons were both significantly lower than previous estimates (rate ratios of 16 and 242 respectively) based on 2002 data for Western Australia [15]. In contrast, our estimates of the age-adjusted rate ratios of chlamydia and gonorrhoea for Aboriginal versus non-aboriginal persons following data linkage remain at least 50 per cent higher than previous estimates for 2004 which were based on the allocation of all cases of unknown Aboriginality to the non-Aboriginal classification [4]. Our use of 2001 census data rather than extrapolations for inter-Census periods, our exclusion of census data with unknown Aboriginality, and our use of 10-year age strata to minimise low cell counts in regression analysis also contribute to differing estimates of age-adjusted rates between this study and other published estimates.

This study only addressed the issue of completeness of identification of Aboriginality. It did not seek to address the issue of validity of identification of Aboriginality. It is possible that Aboriginality as reported in the notification data or any of the linked databases may be inaccurate, and reported Aboriginality is likely to be ascertained by various methods, including through reference to previous medical records, or as an individual judgement based on a person's appearance. The estimation of disease rates based on notification data is also limited by the effect of awareness and screening programs which are often targeted at high-risk groups, and the inclusion of cases associated with contact tracing and follow-up activities as a result of screening or case reports.

The outcomes of this analysis are also limited by the linkage of only one year of notification data with the linked database, and the absence of any additional strategies used to improve the quality of the routinely collected demographic data for notified cases prior to data linkage. The potential benefits of introducing additional strategies to improve data quality and more extensive clerical review processes for pairs of records that represented possible linkages remain to be investigated in future studies; however, the aim of this analysis was to investigate the potential for improving the quality of existing routinely collected data using minimally resource-intensive methods. Further studies linking more recent case notification data, including notifications with known Aboriginality, will offer the capacity to investigate the consistency of the identification of Aboriginality across all data sources, as well as examine trends in the identification of Aboriginality, and Aboriginal and non-Aboriginal disease notification rates over time.

Even disease-specific factors, including the severity of the disease and the need for a rapid public health response have been found to be insufficient to ensure complete reporting of diagnosed cases of notifiable diseases [1]. This study provides evidence to support the benefits of using data linkage for notifiable diseases surveillance within health jurisdictions that have high rates of missing data on Aboriginality, as non-response introduced significant bias in the estimation of disease rates by Aboriginality. Our findings also indicate the importance of introducing strategies to minimise non-response, including item non-response, in the collection of routine disease surveillance data.

Strategies that have been found to improve the completeness of disease reporting include the use of active surveillance, laboratory-notification systems and improved links with clinicians [1]. For routinely collected notifiable disease surveillance data in Western Australia, strategies that are currently being explored to improve the completeness of reporting include the addition of a field identifying Aboriginality on laboratory request forms to allow laboratory-only disease notifications to provide data on Aboriginality, and a message directing doctors to the Western Australian Department of Health's communicable disease notification website [16] which details the procedure for the notification of communicable diseases. Further standardisation of data collection processes in Western Australia may assist in improving the item-level completeness of disease notification data.

For the majority of disease notifications, follow-up to obtain missing information on Aboriginality is not conducted by the Department of Health unless specific public health action is required, as this is a resource-intensive process which requires resources to be withdrawn from other public health activities. In this context, and while strategies are being developed to improve the completeness of reporting, the use of minimally resource-intensive automated data linkage techniques to improve the accuracy of disease notification rates provides an efficient and effective means to improve the quality of routinely collected data and enhance its usefulness. Until the reporting of Aboriginality in disease notification data improves, we recommend that data linkage studies are performed periodically to provide an improved foundation for public health policy.

## Conclusion

The utility of routinely collected disease surveillance data is compromised when a significant proportion of cases have missing data. We used data linkage to reduce substantially the proportion of STI and BBV notifications with missing Aboriginality data. Although there is still a high incidence of STIs and BBVs in Aboriginal people, incompleteness of Aboriginality data contributes to overestimation of the risk associated with Aboriginality for these diseases. Until improvements in the completeness of data collection can be achieved, data linkage provides an efficient method to improve the accuracy of disease notification rates.

## Competing interests

The authors declare that they have no competing interests.

## Authors' contributions

DBM conceived and supervised the study, including the data extraction and linkage components. Both DBM and REW were involved in data analysis, drafting and critically revising the manuscript.

## Pre-publication history

The pre-publication history for this paper can be accessed here:


